# Practical recommendations for addressing the psychological needs of unaccompanied asylum-seeking children in England: A literature and service review

**DOI:** 10.1177/13591045241252858

**Published:** 2024-06-13

**Authors:** See Heng Yim, Glorianne Said, Dorothy King

**Affiliations:** 1 4616 King’s College London, UK; 2 South London and Maudsley NHS Foundation Trust, UK; 3 Foundation 63, UK

**Keywords:** Trauma, displaced, refugee, unaccompanied children, separated children, Traumatic stress, war

## Abstract

**Background:**

Globally, there is an increasing trend of forcibly displaced people, of which over 40% are children. Unaccompanied asylum-seeking children (UASC) are at risk of experiencing psychological distress and developing mental health difficulties. However, in the UK, the approach from statutory mental health services is inconsistent across different geographical areas.

**Aim:**

This report outlines recommendations for statutory mental health services in the UK in relation to working with UASC.

**Method:**

A rapid evaluation method was adopted including interviewing fifteen key informants as well as reviewing existing clinical guidelines. Key informants included clinicians, service managers, social workers and commissioners from Local Authorities, National Health Services, and third sector partners. Recommendations were synthesised using narrative synthesis.

**Results and conclusion:**

Existing service provision and barriers to the implementation of interventions were summarised and compared against existing guidelines. The report presents recommendations on assessments, screening tools, and psychological interventions for developing a pathway for UASC within statutory services.

## Plain language summary

Globally, there is an increasing trend of asylum seekers and refugees, and 40% of which are children. Unaccompanied asylum-seeking children (UASC) are at risk of experiencing psychological distress and developing mental health difficulties. Yet, service provision for this population is inconsistent across different regions in the UK and may not be sufficient to meet their psychosocial needs. We hope to provide recommendations for services in England on how to better support UASC through reviewing the existing literature, clinical guidelines, and interviewing different service providers in the country in order to identify gaps in services. We interviewed key informants, including clinicians, service managers, social workers and commissioners from Local Authorities, National Health Services, and third sector partners. We present findings on how to improve the current assessment, screening and psychological interventions for UASC.

## Introduction

In the UK, children under the age of 18 who are separated from their parents and who are not being cared for by an adult who by law has the responsibility to do so, and who have applied for asylum, are regarded as unaccompanied asylum-seeking children (UASC) (Home Office, 2021). The number of UASC applying for refugee status in the UK increased from 2,399 in 2017 to 4,382 in 2021 (Refugee Council, 2022).

Despite the increasing numbers and elevated prevalence of mental health conditions, primarily traumatic stress reactions and internalising distress (Bamford et al., 2021; Bean et al., 2007), the mental health needs of UASC are not adequately supported in the UK – a high level of underutilisation of statutory mental health services has been found (Mitra & Hodes, 2019). In addition, UASC are placed in several localities across the country following the introduction of the National Transfer Scheme (NTS) in 2016. The NTS was developed to ensure a more equitable number of UASC across local authorities, as many UASC arrive in port cities (Home Office, 2021). This accentuates the importance of a coordinated, consistent approach across services to ensure smooth transitions between services and prevent inequitable access to healthcare across the UK. There is evidence from an expert consensus survey that the National Health Service (NHS) does not currently provide a coordinated approach in providing psychological support to refugees (Munz & Melcop, 2018). Although Munz and Melcop (2018) did not specifically look at provision for child refugees, both client-related and institutional barriers for this population have been found in the UK (Eruyar et al., 2022; Majumder, 2019) and other European countries (Bean, Eurelings-Bontekoe, et al., 2006). In addition, research shows that without appropriate treatment, there is no significant change in mental health symptoms in UASC between arrival and two years following arrival (Jensen et al., 2014).

Given the inconsistencies in the provision of psychological interventions identified in the literature and through reports, this report aimed to (1) review existing recommendations on psychosocial interventions to address UASC mental health needs, (2) examine the current mental health provision within health and social care in England from service providers’ perspectives, and (3) synthesise recommendations and shortcomings in the current approaches to reduce health inequity in statutory settings.

## Method

This project consisted of three phases: (1) a literature review of existing guidelines and reviews on supporting the mental health needs of UASC in England, (2) conducting interviews with service providers (e.g. social workers, clinicians, service managers, commissioners) to understand existing service provision (and barriers to provision) within child and adolescent mental health services and/or social services in England, and (3) a narrative synthesis of recommendations based on the review of the guidelines and interview data (Table 1).

**Table 1.** Summary of Data Collection and Analysis.

Data source	Method of data collection	Inclusion/Participants	Data analysis	Purpose of the data
Guidance documents, published reviews	Literature review on ovid medline, PsycINFO, embase, google scholar via hand search, keyword search, citation search, contacting experts (UASC health, foundation 63, helen bamber foundation/Young roots) for key literature (see S1 on search strategy and search terms)	Inclusion criteria: Guideline documents on UASC mental health, peer-reviewed review articles on psychological interventions for UASC or refugee children in clinical settings in English	Narrative synthesis	To benchmark against existing service provision and to understand the evidence-based psychosocial interventions
Key informant interviews	Thirty-minute semi-structured online video interviews using purposive, convenience and snowball sampling with service providers and researchers between Feb and June 2022 (see S2 for interview topic guide)	Fifteen in total with representation from 10 services Clinical psychologists or systemic psychotherapists in local authority (*n* = 2) Social workers in local authority (*n* = 2) Service manager in CAMHS (*n* = 1) Clinical psychologists or psychotherapists in looked after children team in CAMHS (*n* = 6) Clinical psychologist in third sector working with young refugees (*n* = 1) Independent consultant systemic psychotherapist in UASC work (*n* = 1) Health and social care commissioners (n = 2)	Data were extracted on an ongoing basis using real-time interview notes Emerging findings were discussed with several key informants mid-way in the study and refined throughout Data on service provision were tabulated	To understand best practices, barriers, and gaps in service provision

A rapid evaluation design was used with reference to the rapid evidence synthesis framework from the World Health Organisation (Tricco et al., 2017) and is presented in Table 1. This methodology enabled us to capture the current statutory service provision to inform service development.

Within phase two, verbal informed consent was obtained from service providers prior to interviews. Ethical clearance was not needed as no patient was involved and the report was classified as service evaluation rather than research. The question guide was developed initially with one of the UASC service leads and iteratively after each interview when new themes were identified (Supplemental information S1). The data collection and analysis period lasted for 4 months. Data were analysed directly from the interview notes (Beebe, 2014). Findings from the literature review and interviews were analysed using narrative synthesis, modified from the guidance for systematic implementation reviews (Popay et al., 2006). Data were tabulated and analysed thematically to identify recommendations and barriers to implementation. The themes were generated by SHY, and were then discussed with DK. The results section presents the findings from the review and interviews, and the discussion section presents our recommendations.

## Results

### Literature review of guidance documents and assessment tools

**Figure 1.**
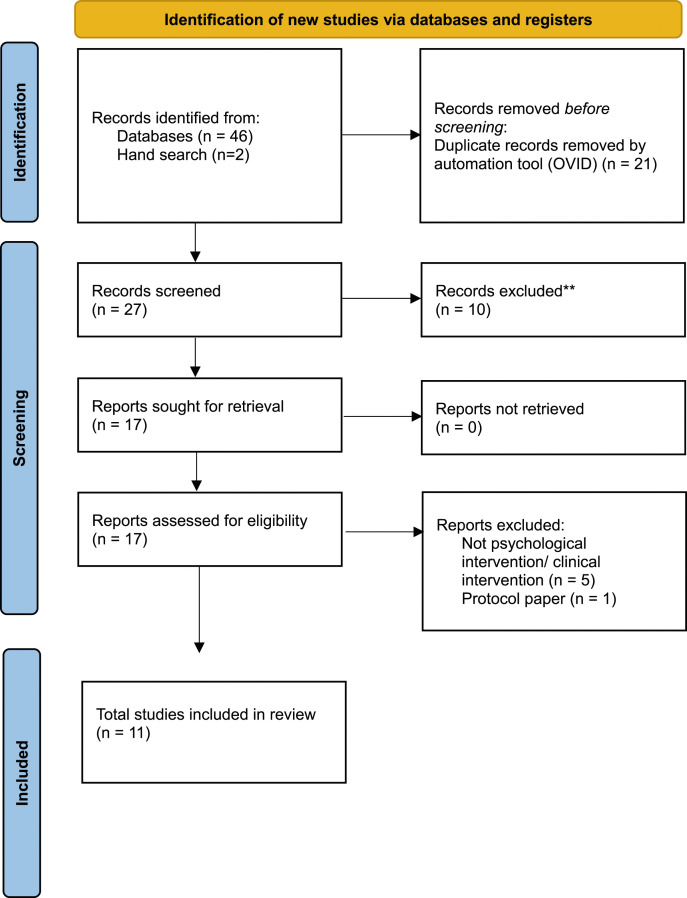
PRISMA flowchart.

S3 shows a list of guidelines and review papers reviewed. Figure 1 shows the PRISMA flowchart of the included reviews. S4 summarises the assessment tools mentioned by the interviewees, and S5 summarises the recommendations of psychosocial interventions from Guidelines and published narrative or systematic reviews. Taken together, guidance documents reviewed recommend providing universal trauma-informed psychosocial care. Screening and assessment are recommended to identify the needs in a timely way (National Institute of Health and Care Excellence, NICE, 2021). However, in terms of screening instruments, very few tools were developed specifically for UASC. Whilst there are validated measures designed for UASC such as the Stressful Life Events (SLE) questionnaire and Reactions of Adolescents to Traumatic Stress Questionnaire (RATS) (Bean, Eurelings-Bontekoe, et al., 2006), they were not validated for DSM-5. Moreover, the Strengths and Difficulties Questionnaire (SDQ) alone is seen as insufficient to assess UASC’s mental health needs (Association of Directors of Children’s Services Ltd, ACDS, 2016; Foundation 63, 2020). A Refugee and Immigrant Core Stressors Toolkit for youth and families has been developed to aid risk assessment (Davis et al., 2021), though the toolkit seems to be more relevant to children arriving with families rather than UASC. Other screening and assessment tools include RHS-15 (Hollifield et al., 2013), Child Revised Impact of Events Scale (CRIES) (Perrin et al., 2005) and the Young Person’s CORE (YP-CORE) (Twigg et al., 2009).

In terms of psychological interventions, most reviews favoured a stepped-care, multi-modal model (e.g. Eruyar et al., 2022; Hodes & Vostanis, 2019) from universal approaches including resilience and skills-building, supporting advocacy, cultural sensitivity (e.g. Foundation 63, 2020; The Children’s Society, 2018; Taylor et al., 2023) to specialist trauma-focused approaches, resembling Herman’s (1992) phased model for recovery from trauma. Creative approaches include play therapy and an expressive art in transition group (Hutchinson et al., 2022), although researchers note that there is currently insufficient evidence for art therapy based on evidence-based practice standards due to the lack of controlled studies (Annous et al., 2022). Trauma-focused approaches described include trauma-focused cognitive behavioural therapy (TF-CBT), CBT, testimonial psychotherapy, narrative exposure therapy (NET), eye movement and desensitisation reprocessing (EMDR), trauma systems therapy, and group teaching recovery techniques (TRT) (Cowling & Anderson, 2023; Hodes & Vostanis, 2019). Based on the NICE guidelines for PTSD, for those that present with a diagnosis or clinically important symptoms of PTSD at least one month after a traumatic event, it is recommended to consider offering an individual TF-CBT intervention for children and young people (NICE, 2018). EMDR is a second-line treatment, which is recommended to be considered if young people do not respond to or engage with a TF-CBT intervention (NICE, 2018). Nevertheless, although the NICE guidelines for PTSD are applicable to asylum seekers and refugees, some systematic reviews have pointed out that there is a lack of randomised controlled trials on the effectiveness of trauma-focused therapies specifically for the UASC population (e.g. Chipalo, 2021; Samarah, 2022).

Other reviews identified some common aims of intervention for UASC, which include building safety, rebuilding identity, coping strategies and social connections/interpersonal skills (e.g. Demazure et al., 2018). Apart from PTSD, other needs such as depression, sleep disturbances, and grief, as well as psychosocial needs such as housing, legal support/advocacy were described (Foundation 63, 2020; The Children’s Society, 2018).

### Existing service provision

Tables 2 and 3 summarise existing support and interventions provided for UASC in the services interviewed. Several interviewees indicated that the pathway development for UASC in their services was recent (in the last 12 months at the time of interview), with the exception of the London services and one service that is located near to a port where the pathways are established.

**Table 2.** Existing Service Provision Through key informant interviews.

Service	Provision	Staffing for psychological/mental health interventions	Interventions provided
1. Child and adolescent mental health service (CAMHS) looked after children (LAC) team in inner-city London	Up to 18 years old. Specialist LAC and refugee team; Co-location with local authority Carried out joint health assessment with another team that works with LAC physical health Used refugee health screening (RHS-15) for screening	Psychiatrists, clinical psychologists (full detail of staffing ratio unknown)	Narrative exposure therapy for children (KIDNET), eye movement desensitisation and reprocessing (EMDR), cognitive behavioural therapy (CBT), teaching recovery techniques (TRT), tree of life No group therapy was running at the time of interview (Feb 2022) Discussion with the team indicates that grounding techniques and stabilisation was the main focus of work rather than trauma-focused therapies
2. CAMHS LAC team in inner-city London	Up to 18 Jointly funded by the NHS and the local authority and co-located with social care Used RHS-15 for screening	0.6 whole time equivalent (WTE) 8a psychologist as UASC lead as of Mar 2022	Mostly stabilisation work Sleep hygiene group had been planned Training with foster carers
3. CAMHS LAC team in southeast england	Up to 18. Sat within LAC pathway within CAMHS.	For the UASC pathway within the LAC team, there was a part-time 0.6 WTE 8b UASC lead art psychotherapist in post	Art psychotherapy, interpersonal therapy, and tree of life group Training with foster carers Consultations to social care staff
4. CAMHS asylum and refugee clinic in southwest england	Up to 18. Specialist team for child refugees	Staffed by a 0.5 WTE clinician from the CAMHS team, 0.1 WTE assistant psychologist and a 0.5 WTE UASC lead art psychotherapist. This structure would be changed with more funding coming in until Mar 2023 for a 0.8 WTE therapy post and a 0.4 WTE assistant psychology post	A rolling group called coping and recovery after trauma experience (CREATE), yearly therapeutic group for foster carers, children accelerated trauma treatment (CATT) and EMDR. UASC were seen for up to 24 sessions There were links with the virtual school once a term and the team lead attended the local UASC meeting with third sector organisations in a monthly forum
5. CAMHS LAC in southeast england	Up to 25. The UASC lead post was commissioned by the local authority though NHS manages the contract Used distress thermometer (The Children’s Society, 2018)	UASC lead was a clinical psychologist. The lead was commissioned to work a day a week (0.2 WTE)	The team ran two stabilisation groups yearly. They follow the “fast feet forward” model (Draper et al., 2020). Psychoeducation on sleep and nutrition, based on materials on UASC health (UASC Health, n.d). Training was available to foster carer
6. CAMHS LAC in southeast england	Up to 25. The team works with them as care leavers until they are 21 or 25 depending on the asylum outcome	A senior clinical psychologist post was commissioned to provide one session a week (consultation or assessment) to social care, and a maximum of 4 sessions (exact WTE unknown)	The psychologist’s time is used in a consultative capacity due to limited time being commissioned UASC were referred to CAMHS LAC team for longer-term work
7. UASC team in local authority in inner city London	Once they turn 18, the UASC is passed on to the LAC team. The affiliated CAMHS LAC team is funded to work with people up to the age of 21. The local authority team can work with them up to the age of 25	A family therapist was the only therapist in the team. However, there were other staff such as social workers, practitioners (work pattern unknown)	Training and facilitated reflective spaces for staff. In terms of therapeutic work, trauma-informed work, nutrition, sleep, and continuing bonds grief therapy (Draper et al., 2020) were offered. There was also a life skills group delivered jointly with CAMHS psychologists Fortnightly systemic therapy clinic for foster families
8. Psychology team in local authority in thames valley	Up to age 25. Pathway currently being set up There was no specialist CAMHS LAC team in the area Screening tool was yet to be decided at the time of interview	Two part-time 8b clinical psychologists and an assistant psychologist (work pattern unknown)	A pilot narrative therapy group (narrative in a suitcase) was being set up provided by an assistant psychologist in the local authority Consultations provided to social care staff
9. A virtual mental health programme pilot in southeast england^1^	A pilot was conducted to embed mentalisation-based psychological support for UASC (Zahra & Chamberlain, 2020, unpublished data)	Six-sessions a week of clinical psychology and five-sessions of an assistant psychologist (exact WTE unknown)	Multi-disciplinary consultation meetings to understand mental health needs of the UASC and provided reflective spaces for staff team
10. Specialist third sector in London	Age group: 11–25	1 senior CP part-time (0.4 WTE), 1 CP part-time (0.4 WTE), one trainee CP (0.2 WTE), and one volunteer AP.	Trauma-specialist individual and group support. Individual work often takes the form of phase 1 and 2 trauma work, as well as psychological support for depression, anxiety, emotional dysregulation, and other difficulties young refugees face. Group is short courses of skills building for trauma related reactions

*Note.* ^1^ The person in charge was unable to be interviewed as they were on maternity leave – they however sent the researchers an evaluation report.

**Table 3.** *Intervention approaches Provided by the services interviewed*.

Theme		Content
Physical health needs	Sleep hygiene and psychoeducation	Three-pronged protocol: Sleep hygiene psychoeducation, a sleep pack (for practical resources such as essential oils), night light and a body clock (documenting and changing sleep pattern) Used to identify when sleep difficulties are due to a nocturnal sleep pattern or trauma symptomology (Carr et al., 2017) An available manual targeting issues faced by UASC is found on UASC health website (https://www.uaschealth.org/resources/mental-health/sleep-eat-hope/)
	Nutrition and refeeding intervention	Clinicians should recognise refeeding syndrome such as gastric reflux and constipation and monitor weight concerns using the medical emergencies in eating disorders (MEED) guidance (Royal College of Psychiatrists, 2022) A refeeding intervention for UASC was developed with reference to the feeding and eating disorders literature (Draper, 2020): Balanced diet and having small meals every 2–3 hours; and support is needed around understanding satiety signals and emotional regulation
PTSD	Narrative exposure therapy for children (KIDNET)	NET is a trauma-focused intervention (Schauer et al., 2011), which includes laying a lifeline using a rope, flowers and stones, and constructing their life story with a focus on the traumatic experiences chronologically, narrating the episodic and sensory details of those events so that exposure and memory and emotional processing is facilitated
	CBT and/or trauma-focused CBT (TF-CBT)	Two individual trauma-focused CBT models in the literature: Cognitive therapy for PTSD (CT-PTSD) (Ehlers & Clark, 2000) and TF-CBT (Cohen et al., 2016) Both include cognitive processing of trauma memory and cognitive reappraisal. In Cohen et al. (2016) model of TF-CBT, it was designed as a family-focused treatment which include parenting skills and conjoint session with carers and children
	EMDR	A trauma-focused therapy where the person focuses on the trauma memory whilst simultaneously experiencing bilateral stimulation (Shapiro, 1995)
Grief	Continuing bonds	A form of enquiry to discuss grief and bereavement, helping people to connect with the deceased in a meaningful way (Klass et al., 2014)
Group interventions	CBT therapy group	A CBT-informed open group to address emotional wellbeing and physical health needs, and introduced resilience-building approaches and coping skills relevant to adjusting to a new social environment (King & Said, 2019)
	Teaching recovery techniques (TRT)	Based on trauma-focused cognitive behavioural therapy developed by the children and war foundation. TRT includes five sessions with young people and two caregiver sessions to increase coping and promote recovery (Yule et al., 2013)
	Narrative therapy (tree of life group, narratives in a suitcase)	Tree of life (Ncube, 2006) Rather than focusing on the problem-saturated narrative (about the trauma), the intervention uses trees as a metaphor and opens up stories about roots (people’s culture and history), ground (present life), trunk (skills and abilities), branches (hopes and goals), and fruits/leaves (gifts from people/important people), encouraging exploration of hopes, skills and goals Narrative in a suitcase Based on narrative therapy by the same author that uses a journey metaphor to support child refugees. The children are invited to create a suitcase, then tell their stories (e.g. special memories, important people, and reasons to leave, things that left behind) in a story circle. They then create a map of their journey, and a banner is made as a documentation of the collective stories
	Fast feet forward	A therapeutic group approach drawn from sports research and the bilateral movements aspects of EMDR (Draper et al., 2020) using feet movements (i.e. running) to support trauma processing whilst narrating trauma ‘hot spots’. Sport coaches deliver the group alongside social workers and a therapist for 1 hour two times a week
	Children accelerated trauma treatment (CATT)	An integrative trauma-focused therapy that integrates cognitive behavioural therapy, creative arts and human rights (Raby & Edwards, 2011). There are 12 stages in the treatment and some components include psychoeducation, imagery, and traumatic episodes are retold using characters from craft materials
Training/consultation work		Clinicians provide training, reflective practice and consultation to GP, social workers, hostel staff, and foster carers. The content includes psychoeducation on trauma and trauma-informed care, cultural understandings of trauma, attachment theory, managing challenging behaviours and aggression, communication breakdown and placement/relationship breakdown, as well as burnout/vicarious trauma

### Barriers and facilitators identified by key informants

Implementation barriers are classified into commissioning challenges and system-related challenges.

#### Commissioning challenges

One commissioner interviewed indicated that as numbers of UASC in the county are low, they did not review CAMHS access as this was not a commissioning priority. Moreover, at the time of the interview, whilst social care received funding for UASC from the NTS, CAMHS teams do not receive additional funding for UASC. Additional problems related to the NTS include that children can be relocated to other counties at short notice, affecting the continuity of service access. Another commissioner expressed concern from clinicians who felt uncomfortable delivering trauma-focused treatments when young people might be returning to their residences with no support, as most UASC lived in shared accommodation rather than with foster families. Therefore, the commissioner expressed a preference for commissioning third sector ‘light-touch therapeutic input’ over trauma-focused interventions.

#### System-related challenges

A social worker described logistical challenges for UASC to get to appointments or for social workers to do home visits as young people resided in different areas and some placements were out of the county. Another social worker expressed concerns about the high eligibility threshold for CAMHS in the local area, and that referrals were being rejected due to not fitting into the Eurocentric diagnostic criteria of mental health difficulties. The interviewee gave an example of unaccompanied minors expressing their distress in ways that does not fit within Western diagnostic frameworks (e.g. they may complain about headaches or not sleeping well), and do not use commonly used diagnostic phrases such as experiencing anxiety or depression within mental health services in the UK. Additionally, the third sector provider shared that mainstream services do not work with young people beyond 18, age disputes then pose a significant barrier to UASC accessing appropriate services. Another challenge relates to monitoring of data, where a commissioner mentioned the number of UASC seeking services were difficult to track and monitor as only looked after children status but not asylum status was recorded in some databases.

#### Facilitators

Interviewees described multi-agency working and co-location with social care/health staff helpful in supporting UASC. Some services expressed linking up with social workers, education pastoral care, key workers in accommodation sites, and third sector organisations and having regular network liaison being the ‘best practice’. One of the commissioners described having good foster carers could facilitate their integration and improve their mental health, and would involve foster carers in the procurement of services.

## Discussion

### Narrative synthesis of service recommendations

Different models of service and commissioning arrangements were implemented in the services interviewed including specialist looked after children mental health teams, community CAMHS, and psychology provision within social care (Table 2). Some only offer psychoeducation and stabilisation groups, some also offer specialised psychological interventions, whereas some offer consultations or reflective spaces only. Interventions provided in the services were heterogeneous (Table 3). Comparing the current services included in this report against existing the guidelines and published reviews, some services fell short of providing flexible entry into mental health services, outreaching in service promotion and delivery, and implementing evidence-based practice, as many services did not have the resources/staffing to provide individual therapy sessions. Some interviewees expressed hesitance in providing trauma-focused interventions due to worries that UASC would not have enough support at their residences.

The section below synthesises recommendations for better supporting UASC in local services.

### Assessment

A comprehensive assessment including screening and assessing physical, psychosocial, mental health and neurodevelopmental needs and risks is needed in the context of young people’s school and community environment. It is recommended that a mental health-trained clinician joins the LAC health assessment. Apart from the SDQ (Goodman, 1997) that is required by the local authorities for all LAC, the Refugee Health Screen (RHS-15) is a commonly used screening measure for emotional distress (anxiety, depression and PTSD) in CAMHS LAC teams interviewed in this report (Hollifield et al., 2013). It is suitable for administration from aged 14 and above, unlike the SDQ where there is an age cut-off of 17. The developers of the RHS-15 tool caution against only using this as an early screening immediately after arrival as emotional distress might be delayed (Hollifield et al., 2013). A modified version of the tool, the distress thermometer, is also used in CAMHS services interviewed in this report (The Children’s Society, 2018). In addition to administering SDQ to under 17, it is recommended to screen all UASC for PTSD. The Child Revised Impact of Events Scale (CRIES) can be used to screen for PTSD, alongside clinical interview, for those who are aged between 8–18. Clinicians need to be aware of the limitations of these measures such as differences in linguistics or cultural beliefs and expressions of distress.

Some UASC find the NHS assessment process unpleasant due to the focus on asking trauma history (Dubs et al., 2022). It is therefore important to focus on engagement in the initial assessment. Trauma history may not need to be discussed in detail at this point particularly if the trauma is already known from the records. It is argued that assessment of pattern and quality of sleep is a good proxy measure of UASC’s mental health as it is less culturally-laden (Bronstein & Montgomery, 2013). The BEARS screening tool for sleep assesses Bedtime, Excessive daytime sleepiness, Awakening during the night, Regularity and duration of sleep, and Snoring (Owens & Dalzell, 2005), though this tool has not yet been validated. When using screening tools, clinicians should be culturally sensitive and follow a trauma-informed approach.

In addition to usual risk and safeguarding assessments, assessors need to be aware of signs of trafficking as well as the impact of human trafficking, where control from abusers/traffickers may persist beyond direct contact through various means including cultural practices such as juju (Foundation 63, 2020), and continued attachment relationships to their traffickers.

### Psychological interventions

Although reviews pointed out the lack of RCTs on the effectiveness of trauma-focused therapies for UASC, they should not be denied NICE-recommended interventions. Evidence-based treatments should be provided based on their presenting complaints. Reviews also identified common treatment goals, including facilitating a sense of safety, rebuilding identity, emotional coping, and social connections.

### Diversity and inclusion considerations

#### Age

The age cut-off (18 years old) in many CAMHS services pose a barrier to access. In 2021, over 78% of UASC applying for asylum in the UK were aged 16 or over (Refugee Council, 2022). However, age assessments and disputes are common and result in vulnerable young people being excluded from both CAMHS and adult services whilst assessments are ongoing (The Children’s Society, 2018). To ensure consistency of support and enable access to age disputed young people, it would be helpful if the upper age range for CAMHS LAC can be raised to 21 or 25.

#### Culture

NICE guidelines state that the costs incurred for the use of professional interpreters are justified and mandated by statutory guidance and remote delivery should be considered to improve access (NICE, 2021). Regional accents and dialects should be taken into consideration when allocating interpreters. Cultural sensitivities need to be considered at all stages of contact (assessment, consultation, and therapy), and culturally-adapted and translated resources should be used where possible. However, UASC may not be literate and translated sources may not always be appropriate. Preferences should be discussed directly with the young person and decided on a case-by-case basis.

UASC may not be familiar with regular appointments or understand how to travel to clinics. Social workers are recommended to support session booking and communicate about upcoming appointments to help facilitate engagement.

### System-related recommendations

#### Consistent and accurate recording for monitoring purposes

It is recommended that the numbers and needs of UASC are included in each County’s Joint Strategic Needs Assessment for monitoring. A recommended minimum dataset has been suggested by UASC Health (n.d.) (https://www.uaschealth.org/wp-content/uploads/2017/02/quality-markers.pdf) recommended by an interviewee. It is recommended that UASC’s asylum status should be recorded for monitoring.

#### Partnership working and the development of a network

Co-location of CAMHS UASC clinicians and the social care LAC team was seen as helpful for integrated working and relationship-building through ‘corridor and informal chats’. One way to encourage an integrated approach is to set up regular consultation slots, joint team meetings and to place a CAMHS clinician and a practitioner from social care who may co-lead training and groups. An ‘outreach’ approach may be needed for the CAMHS clinician to go to reception centres or supported accommodation to deliver groups and trainings to facilitate engagement (Foundation 63, 2020). The stakeholders in the local area include solicitors, faith leaders, foster parents, looked after children health teams, schools and virtual schools, third sector organisations, GPs, CAMHS LAC teams, local authority LAC teams, adult mental health teams (to ensure smooth transitions between services). When needed, the young person is recommended to be signposted to Red Cross Family Tracing. Mental health-trained clinicians are able to provide attachment-informed, trauma-informed trainings and consultations to other stakeholders and to facilitate multi-agency working.

### Commissioning

Given the rising numbers of UASC coming into Local Authority care, more funding is needed. This is in line with the NHS Long-term plan (trauma-informed care) as well as the commitment to increase funding to improve and expand access to care for children and young people (NHS, 2019). However, money from the National Transfer Scheme goes to social care only, not to CAMHS. Commissioning, particularly with the introduction of integrated care systems, should promote joint funding for a more holistic, wrap-around support. It is also clearly written in the NICE guideline that the costs incurred for professional interpreters are justified and longer sessions may be needed (NICE, 2021).

**Figure 2.**
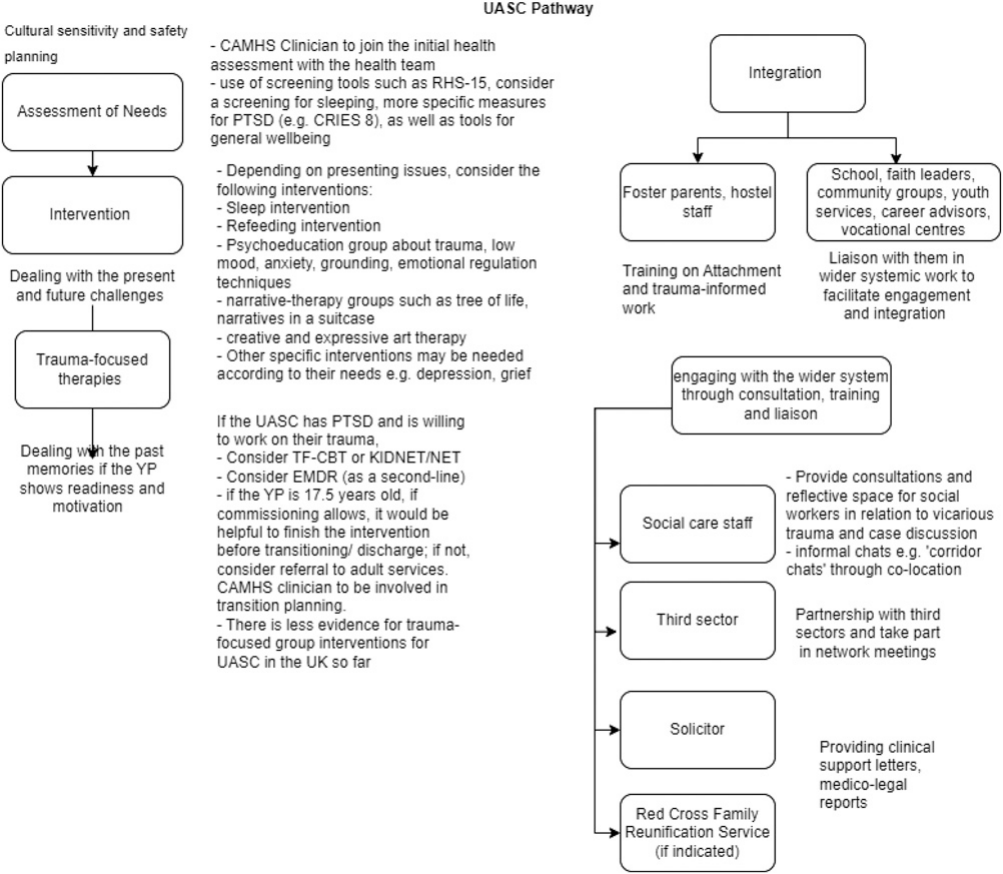
Proposed UASC service provision.

Figure 2 shows a proposed UASC service provision based on the synthesised results. We outline our suggestions based on synthesising existing guidelines, reviews, and interviewees’ responses. The pathway includes types of assessment and interventions offered, as well as links with other agencies to facilitate integration and interdisciplinary working. We envision this as a ‘living’ pathway model given the field is evolving.

### Limitations and future research

As a pragmatic report that adopted a rapid evaluation and synthesis methodology, certain perspectives were missing, namely the lived experience perspectives of foster carers and UASC. Interviews with stakeholders were 30 minutes only, as it was not feasible for interviewees to dedicate more time amidst their busy clinical work. The report also lacks representation from adult specialist NHS services such as traumatic stress services working with refugees and asylum seekers. This report can however act as a basis for a more thorough service and workforce mapping exercise in the future, including making Freedom of Information requests to different councils and services across England, or a Delphi study looking at consensus for best practice of working with UASC.

There is a lack of reports and studies showing routine clinical delivery for UASC. Whilst developing the local mental health pathway for UASC, it is recommended to demonstrate and evaluate the effectiveness of the work for sharing good practices.

In terms of future research, a useful research question might be to explore whether there is a difference between effectiveness of treatments when UASC are placed in foster placements versus semi-independent living. More research is needed to ascertain the effectiveness of different psychological interventions for UASC in the UK as reviews reported a lack of RCTs to support specific interventions for this group. Another area that is crucial but not discussed in length in this evaluation are the systemic factors (notably immigration policy and asylum status) that impact on UASC’s mental health and the clinician’s role, such as liaison with immigration solicitors. Based on the current findings, although we did not specifically ask about the legal needs, interviewees did not describe the impact of immigration policy or clinician’s role in supporting their legal needs (e.g. writing medicolegal reports, supporting letters). Further research is needed to understand the perspectives of immigration lawyers on how mental health clinicians can support the legal needs of UASC.

In conclusion, the report sheds light on the existing service gaps for UASC. Unique challenges with this population were discussed, including age limits in accessing CAMHS and age disputes, responding to cultural needs (e.g. how mental health is understood, use of interpreters), as well as clinicians’ concerns around commencing trauma-focused therapies. It also highlights important areas of recommendations (e.g. flexible modes of assessment and entry to the service, assessments, interventions, diversity considerations and adaptations, system considerations) for service providers when developing a UASC pathway.

## Supplemental Material

Supplemental Material - Practical recommendations for addressing the psychological needs of unaccompanied asylum-seeking children in England: A literature and service review Supplemental Material for Practical recommendations for addressing the psychological needs of unaccompanied asylum-seeking children in England: A literature and service review by See Heng Yim, Glorianne Said and Dorothy King in Clinical Child Psychology and Psychiatry.
