# Supplementary material for: Practical recommendations for addressing the psychological needs of unaccompanied asylum-seeking children in England: A literature and service review
Source: Clin Child Psychol Psychiatry. 2024 Jun 13;30(2):245–63. doi: 10.1177/13591045241252858 (PMC11951383; doi:10.1177/13591045241252858)
Supplement: Supplemental Material - Practical recommendations for addressing the psychological needs of unaccompanied asylum-seeking children in England: A literature and service review [file sj-pdf-1-ccp-10.1177_13591045241252858.pdf]

Supplementary information

S1. Search strategy and search terms

Search strategy: Hand search and database search

Embase <1974 to 2024 February 05>
Ovid MEDLINE(R) ALL <1946 to February 05, 2024>
APA PsycArticles Full Text

Search terms:

Variations of UASC: unaccompanied asylum seeking children, unaccompanied refugee minors, unaccompanied asylum seeking minors, refugee children, refugee youth, refugee adolescent, UASC, UAM

Variations of review: scoping review, systematic review, literature review, practitioner review, review

Variations of interventions: psychological intervention, intervention, therapy, psychosocial intervention, psychological treatment, psychosocial treatment, group intervention, school-based intervention

S2. Interview Questions

(1) What kind of interventions are you offering for UASC?
What's on offer in your respective CAMHS team/ local authority/ area (commissioner)?
Is there a specific mental health pathway within the CAMHS/local authority service?
(2) What does the model look like in your area (ie. The collaboration between social care/ local authority, CAMHS and the Refugee council/ third sector etc?)
(3) Age range your team supports
(4) When are they supported by CAMHS LAC team, and when will they be supported by general/ community CAMHS?
(5) What are the specific screening measures used for UASC?
(6) What are the key guidance documents you used for your role?
(7) Barriers, challenges and facilitators in your work with UASC
(8) Anything not covered but you would like to talk about (e.g. funding)

Commissioner:
1. What are the needs in the area in terms of UASC support?
2. How've the needs changed over the years?
3. What's provided in your respective CAMHS team/ local authority/ area (commissioner)?
4. What are the commissioning priorities?
5. Gaps in providing mental health support in the area?
6. Barriers identified as a commissioner

S3. Guidelines and Review Articles reviewed

Guidance documents

- Association of Directors of Children's Services Ltd. (ADCS) (2016). Safeguarding Pressures Phases 5- Special Thematic Report on Unaccompanied Asylum Seeking Refugee Children. https://adcs.org.uk/assets/documentation/ADCS_UASC_Report_Final_FOR_PUBLICATION.pdf
- Foundation 63 (2020). Practice guidelines for clinical psychologists for supporting appropriate care and treatment for Unaccompanied Asylum-Seeking Minors in the United Kingdom. https://acpuk.org.uk/wp-content/uploads/2020/01/Guidelines-for-working-with-UAM-1.pdf
- National Institute for Health and Care Excellence. (2018). Post-traumatic stress disorder NICE Guideline [NG116]. https://www.nice.org.uk/guidance/ng116/chapter/Recommendations#management-of-ptsd-in-children-young-people-and-adults
- National Institute for Health and Care Excellence (2021). Looked-after children and young people NICE guideline [NG205]. https://www.nice.org.uk/guidance/ng205
- The Children's Society (2018). Distress signals: unaccompanied young people's struggle for mental health care. https://www.childrenssociety.org.uk/what-we-do/resources-and-publications/distress-signals

Review articles

- Annous, N., Al-Hroub, A., & El Zein, F. (2022). A Systematic Review of Empirical Evidence on Art Therapy With Traumatized Refugee Children and Youth. *Frontiers in Psychology*, *13*. https://doi.org/10.3389/fpsyg.2022.811515
- Chipalo, E. (2021). Is Trauma Focused-Cognitive Behavioral Therapy (TF-CBT) Effective in Reducing Trauma Symptoms among Traumatized Refugee Children? A Systematic Review. Journal of Child & Adolescent Trauma, 14(4), 545–558. https://doi.org/10.1007/s40653-021-00370-0
- Cowling, M. M., & Anderson, J. R. (2023). The effectiveness of therapeutic interventions on psychological distress in refugee children: A systematic review. *Journal of Clinical Psychology*, *79*(8), 1857–1874. https://doi.org/10.1002/jclp.23479
- Demazure, G., Gaultier, S., & Pinsault, N. (2018). Dealing with difference: A scoping review of psychotherapeutic interventions with unaccompanied refugee minors. European Child & Adolescent Psychiatry, 27(4), 447–466. https://doi.org/10.1007/s00787-017-1083-y
- Ehntholt, K.A. and Yule, W. (2006), Practitioner Review: Assessment and treatment of refugee children and adolescents who have experienced war-related trauma. Journal of Child Psychology and Psychiatry, 47: 1197-1210. https://doi.org/10.1111/j.1469-7610.2006.01638.x
- Eruyar, S., Huemer, J. and Vostanis, P. (2018), Review: How should child mental health services respond to the refugee crisis?. Child Adolesc Ment Health, 23: 303-312. https://doi.org/10.1111/camh.12252
- Hodes, M. and Vostanis, P. (2019), Practitioner Review: Mental health problems of refugee children and adolescents and their management. J Child Psychol Psychiatr, 60: 716-731. https://doi.org/10.1111/jcpp.13002

- Hutchinson, R., King, N., & Majumder, P. (2022). How effective is group intervention in the treatment for unaccompanied and accompanied refugee minors with mental health difficulties: A systematic review. *International Journal of Social Psychiatry*, *68*(3), 484–499. https://doi.org/10.1177/00207640211057727
- Oberg, C., & Sharma, H. (2023). Post-Traumatic Stress Disorder in Unaccompanied Refugee Minors: Prevalence, Contributing and Protective Factors, and Effective Interventions: A Scoping Review. *Children*, *10*(6). https://doi.org/10.3390/children10060941
- Samarah, E. M. S. (2022). Narrative exposure therapy to address PTSD symptomology with refugee and migrant children and youth: A review. *Traumatology.* Advance online publication. https://doi.org/10.1037/trm0000427
- Taylor, A., Radford, G. and Calia, C. (2023), Review: Cultural adaptations to psychosocial interventions for families with refugee/asylum-seeker status in the United Kingdom – a systematic review. Child Adolesc Ment Health, 28: 241-257. https://doi.org/10.1111/camh.12547

S4. Assessment tools mentioned by interviewees

Tool	Reference	Areas screened or assessed	Age
The Refugee and Immigrant Core Stressors Toolkit (RICST)	Davis, S. H., Winer, J. P., Gillespie, S. C., & Mulder, L. A. (2021). The Refugee and Immigrant Core Stressors Toolkit (RICST): Understanding the Multifaceted Needs of Refugee and Immigrant Youth and Families Through a Four Core Stressors Framework. Journal of Technology in Behavioral Science, 6(4), 620–630. https://doi.org/10.1007/s41347-021-00218-2.	Stressors include trauma, acculturative stress, isolation, resettlement	Not mentioned
Strengths and Difficulties Questionnaire (SDQ)	Goodman, R. (1997). The Strengths and Difficulties Questionnaire: A Research Note. Journal of Child Psychology and Psychiatry, 38(5), 581–586. https://doi.org/10.1111/j.1469-7610.1997.tb01545.x	Emotional symptoms, conduct problems, hyperactivity, peer problems and prosocial behaviour based on DSM 5	4-17 years old
Refugee Health Screener-15 (RHS-15)	Hollifield, M., Verbillis-Kolp, S., Farmer, B., Toolson, E. C., Woldehaimanot, T., Yamazaki, J., Holland, A., St. Clair, J., & SooHoo, J. (2013). The Refugee Health Screener-15 (RHS-15):	Emotional distress (anxiety, depression	Aged 14 or above

	Development and validation of an instrument for anxiety, depression, and PTSD in refugees. General Hospital Psychiatry, 35(2), 202–209. https://doi.org/10.1016/j.genhosppsych.2012.12.002	and PTSD)	
‘BEARS’ sleep screening tool	Owens, J. A., & Dalzell, V. (2005). Use of the ‘BEARS’ sleep screening tool in a pediatric residents’ continuity clinic: A pilot study. Sleep Medicine, 6(1), 63–69. https://doi.org/10.1016/j.sleep.2004.07.015	Sleep	Not validated
Child Revised Impact of Events Scale (CRIES)	Salari, R., Malekian, C., Linck, L., Kristiansson, R., & Sarkadi, A. (2017). Screening for PTSD symptoms in unaccompanied refugee minors: A test of the CRIES-8 questionnaire in routine care. Scandinavian Journal of Public Health, 45(6), 605–611. https://doi.org/10.1177/1403494817715516 A link to various translations of CRIES is here: https://www.childrenandwar.org/measures	PTSD (DSM-IV)	8-18 years old
The Young Person’s CORE (YP-CORE)	Twigg, E., Barkham, M., Bewick, B.M.m Mulhern, B., Connell, J. & Cooper, M. (2009) The Young Person's CORE: Development of a brief outcome measure for young people. Counselling and Psychotherapy Research, 9 (3). https://doi.org/10.1080/14733140902979722	A session-by-session monitoring measure that covers anxiety, depression, trauma, physical problems, functioning and risk to self	11-16 years old

S5 – Summary of recommendations from guidelines and reviews

Guidelines	
ADCS, 2016	- Authorities new to receiving UASCs found it helpful to get support from experienced local authorities<br>- Specialist knowledge around immigration law, use of interpreters, Merton age assessments<br>- A review of 154 initial health assessments identified the following mental health needs: PTSD, depression and sleep disorder
Foundation 63, 2020	- There is high prevalence of PTSD in this population and PTSD should be screened for/considered. However, we should caution against assuming that all UASC suffer from PTSD.<br>- Effective engagement, supporting flexible entry into mental health services, multi-agency work<br>- Using well-trained interpreters where needed<br>- Careful of the ongoing risks of control the UASC experience such as contacts with trafficker<br>- Mental health needs: identify needs through routinely screening for difficulties using standardised measures; offering evidence-based interventions; using coproduced care plans to support socio-political needs; supporting access to trauma-focused interventions; supporting UASC with asylum-related stress<br>- Physical health needs: assessment and treatment of physical health needs in appropriate services; advocate for the sensitive delivery of physical health interventions<br>- Legal needs: awareness of the asylum process; liaison with solicitors as part of multi-agency working; provision of support/ advocacy through professional letters<br>- Social needs: access to education, liaison with advocacy and third sector organisations, promote involvement in social activities
PTSD guideline (NICE, 2018)	- Use interpreters or offer a choice of therapists if language or culture differences present challenge to psychological therapies in PTSD<br>- For prevention of PTSD, a group trauma-focused CBT intervention for children and young people aged 7 to 17 years can be considered if there has been an event within the last month leading to large-scale shared trauma<br>- For those that present with trauma symptoms at least a month after a traumatic event, NICE guidance for PTSD recommends considering and offering trauma-focused CBT (TF-CBT) for the young person (NICE, 2018). There is less evidence for trauma-focused group interventions so far.<br>- EMDR is a second-line treatment if they do not respond to or engage with TF-CBT (NICE, 2018).<br>- For those with complex PTSD, build in extra time to develop trust, take into account of the person's safety and stability, manage barriers to treatment engagement, work together and plan ongoing support after treatment.

LAC guideline (including UASC) (NICE, 2021)	- Support positive relationships in the care network<br>- Aware of the issues that affect this group, including health needs, safeguarding issues, language and culturally sensitive care needs, and the danger of going missing<br>- Aware that they may face additional disadvantage and ensure their needs are met and do not face further marginalisation<br>- Offer culturally appropriate, tailored initial health assessment that address risks arising from their country of origin and journey to the UK which include diet and nutrition, gastrointestinal symptoms, oral health, immunisation status, tuberculosis screening, sexual health, infectious disease, sensory issues, mental health, sleep disturbances
The Children's Society, 2018	- More comprehensive tools for assessing mental health need are needed; mental health needs identified include effects of trauma, sleep disturbances, access to education, bereavement, a lack of secure accommodations/ placements, social isolation, self-harm and suicide, age assessments also can create mental health distress which lead to their identities being challenged or inability to access children's services<br>- Provide advocacy support<br>- Conduct assessments and determinations in the UASC's best interests<br>- More connected services and effective leadership to ensure joint working<br>- Targeted trainings to improve awareness and identification of needs<br>- Support with family reunion<br>- Having a sensitive, adaptable, community-based referral process<br>- Building relationships and trust with UASC<br>- Provide a range of adaptative resources (format, language) to enable them to communicate their mental health needs
Reviews	
Annous et al., 2022	- Systematic review on art therapy with traumatised refugee children on PTSD between 2010 and 2020. Eight papers were included. The authors concluded that there was insufficient evidence for art therapy at the moment based on the Council for Exceptional Children (CEC) evidence-based practice (EBP) standards. The majority of the studies were with no control group and other times art therapy were used as an adjunct to other forms of therapy.
Cowling & Anderson, 2023	- Systematic review on the effectiveness of psychological interventions on distress amongst refugee children. Seventy-one quantitative articles were identified. Interventions include CBT (n=16), NET (n=13), psychosocial interventions (n=13), expressive art therapy (EAT) (n=13), multimodal interventions (n=6), family/parent-focused interventions (for the accompanied minors) (n=8), EMDR (n=5), play therapy (n=3), trauma systems therapy (n=1) and positive psychology interventions (n=1).<br>- Whilst there are positive findings for a variety of interventions reviewed, the authors argued that CBT (and its variations) has the

	largest evidence base within this body of research in reducing psychopathologies such as PTSD and depression.
Chipalo, 2021	- Four studies were included to examine the effectiveness of TF-CBT in reducing trauma symptoms among child refugees. Reduction in trauma symptoms were found but the studies were of pilot nature. The included studies were conducted in the US and in Germany.
Demazure et al., 2018	- This is a scoping review on psychotherapeutic interventions with URM.<br>- They were unable to conclude or recommend specific interventions, but they summarised the aims of interventions. These include creating a safe place, rebuilding identity, developing coping strategies, resilience, emotional regulation, improving self-esteem and social connections. They did not find whether individual or group intervention was more superior.
Ehntholt & Yule, 2006	- This is a practitioner review, rather than a systematic review. They focused on war-affected children rather than only UASC<br>- A phased model of intervention was proposed although the authors mentioned this reflects a pragmatic approach rather than based on research evidence<br>- Promising treatments for war-related PTSD suggested were CBT, testimonial psychotherapy, NET, and EMDR<br>- Spiritual leaders, rituals and traditional healers may be appropriate with regard to traumatic grief<br>- Research into interventions for war-affected children under the age of 8 was scarce.<br>- Good knowledge of working with interpreters, USAC-specific needs, cross-cultural differences are needed<br>- Medico-legal report writing was seen as a skill needed for professionals working with young refugees
Eruyar et al., 2017	- Systematic review and synthesis of interventions in high-income countries for refugee children included predominantly trauma-focused interventions with varied theoretical frameworks, including cognitive behavioural, exposure, narrative, testimonial, interpersonal, reprocessing, eye moment desensitisation and creative therapies.<br>- The authors proposed a multimodal approach tailored to the child's situation and psychosocial needs. The model includes six levels:<br>Level 1: safety (environment, attitudes)<br>Level 2: Nurturing (family, carers)<br>Level 3: Resilience-building (school, community)<br>Level 4: Applications of therapeutic approaches<br>Level 5: Counselling/ psychological interventions<br>Level 6: Access to mental health services
Hodes & Vostanis, 2018	- This is a practitioner review, rather than a systematic review. They focused on refugee children in general rather than UASC<br>- The authors argued for that understanding refugee children's service activity patterns and reasons behind the patterns are important

	- They proposed a hierarchy of interventions from enhancing children's sense of safety, coping strategies and adaptive functioning through psychoeducation and skills-based programmes (e.g. problem-solving) before trauma-focused interventions<br>- Approaches discussed in the review include CBT, NET, EMDR, and group TRT programme, however, authors acknowledged that randomised controlled trials were limited
Hutchinson et al., 2022	- This systematic review examined the effectiveness of group interventions for both UAMs and accompanied refugee minors (ARMs). Seventeen papers (qualitative, quantitative, mixed methods) were included and demonstrated an improvement of mental health outcomes although the authors also concluded that most studies did not have a robust methodology (three RCTs). The group interventions that were evaluated in RCTs include My Way (or Mein Weg in German), Writing for Recovery group and Expressive Arts in Transition (EXIT) group<br>- My Way is a component-based intervention combining TFCBT and group-processing.<br>- EXIT is an intermodal expressive art therapy with music education and music/art/dance therapy following Herman (1992) phase one principles on stabilisation<br>- Mixed method studies included use of group EMDR and group TF-CBT, mindfulness-based intervention, and TRT<br>- The researchers called for more studies on comparing the effectiveness of individual versus group interventions
Oberg & Sharma, 2023	- This is a scoping review on the prevalence, contributing and protective factors as well as interventions for PTSD in unaccompanied refugee minors (URM). Five interventions were included: TFCBT, My Way or 'Mein Weg', TRT, KIDNET (NET for children), and expressive arts intervention (EXIT). As this is a scoping review, the focus was not on effectiveness.<br>- The authors asserted that the results for TFCBT was promising but more research is needed specifically on URM population.<br>- My Way (Mein Weg in German) - There was one RCT (Pfeiffer et al., 2018) with 50 male participants and found that the group intervention was effective in reducing PTSD and depression.<br>- For TRT, a RCT was reported to be underway and another study demonstrated TRT could enhance life satisfaction.<br>- EXIT - There was one controlled study on male URM, and the results demonstrated reductions in PTSD symptoms, as well as a greater sense of safety, calming, connectedness, community efficacy, hope.<br>- The authors identified two RCTs on KIDNET, one in Finland and one in Germany on URM and PTSD.
Samarah, 2022	- Four studies were included on the use of NET among refugee children who experienced multiple trauma in Sri Lanka, Germany and Finland. Significant between-group effects were found in two of the four studies. The author conclude that there were limited RCTs

	on this topic.
Taylor et al., 2022	- The systematic reviewed cultural adaptations to psychosocial interventions for refugee families in the UK rather than UASC<br>- Eleven studies were included, however they were not able to compare the effectiveness of culturally-adapted interventions versus non-culturally adapted ones<br>- Adaptations included interpreters, consideration of their developmental and literacy level, using translated materials, delivered by native community workers, inclusion of traditional music, food, games, cultural values, cultural-specific symbols, delivered in a safe location. Intervention content targeted specific cultural difficulties e.g. asylum process, use of cultural-specific outcome measures, acknowledgement of their loss, sensitivity to stigma

References

Herman, J. L. (1992). Trauma and recovery. Basic Books

Pfeiffer, E., Sachser, C., Rohlmann, F., & Goldbeck, L. (2018). Effectiveness of a trauma-focused group intervention for young refugees: A randomized controlled trial. Journal of Child Psychology and Psychiatry, 59(11), 1171–1179. https://doi.org/10.1111/jcpp.12908
